# Capecitabine-associated gastrointestinal ulceration, haemorrhage, and obstruction: a pharmacovigilance analysis based on the FAERS

**DOI:** 10.3389/fphar.2024.1412938

**Published:** 2024-06-14

**Authors:** Yuwei Wang, Long Meng, Xiao Liu

**Affiliations:** ^1^ Department of Radiation Oncology, The Cancer Hospital of Chongqing University, Chongqing, China; ^2^ Department of Pharmacy, The First Affiliated Hospital of Chongqing Medical University, Chongqing, China; ^3^ Department of Gastrointestinal Surgery, The Fifth People’s Hospital of Chongqing, Chongqing, China

**Keywords:** adverse drug reactions, capecitabine, FAERS, gastrointestinal ulceration, gastrointestinal haemorrhage, gastrointestinal obstructions

## Abstract

**Background:**

Capecitabine has been reported to be associated with severe gastrointestinal (GI) adverse drug reactions (gastrointestinal ulceration, haemorrhage, and obstruction). However, statistical correlations have not been demonstrated, and specific GI adverse drug reactions, such as GI obstruction, are not listed on its label.

**Aim:**

We aimed to determine the associations between capecitabine and GI ulceration, haemorrhage, or obstruction among patients with breast cancer by examining data from the United States Food and Drug Administration Adverse Event Reporting System (FAERS).

**Methods:**

We performed disproportionality analysis of GI ulceration, haemorrhage, and obstruction by evaluating the reporting odds ratio (ROR) and the information component (IC) with their 95% confidence intervals (CIs).

**Results:**

We identified 279 patients with capecitabine-associated GI ulceration, haemorrhage, or obstruction reported between 1 January 2004 and 31 December 2020. One-fourth of the cases of GI ulceration, haemorrhage, or obstruction resulted in death. Capecitabine as a drug class had disproportionately high reporting rates for GI ulceration [ROR 1.94 (1.71–2.21); IC 0.80 (0.60–0.99)], haemorrhage [ROR 2.27 (1.86–2.76); IC 0.99 (0.69–1.28)], and obstruction [ROR 2.19 (1.63–2.95); IC 0.96 (0.51–1.40)].

**Conclusion:**

Pharmacovigilance research on the FAERS has revealed a slight increase in reports of GI ulceration, haemorrhage, and obstruction in capecitabine users, which may cause serious or deadly consequences. In addition to the adverse reactions described in the package insert, close attention should be paid to GI obstruction to avoid discontinuation or life-threatening outcomes.

## Introduction

Intravenous 5-fluorouracil (5-FU) is an antimetabolite chemotherapeutic agent that requires hospitalization for constant infusion through a peripheral catheter or, if the venous condition is poor, a central catheter. By comparison, oral capecitabine, a fluoropyrimidine carbamate, is quickly absorbed through the intestine and converted to 5-FU by the enzyme thymidine phosphorylase, thereby inhibiting the ability of cancer cells to produce substances necessary for growth (Saif et al., 2008). Capecitabine, which is an effective and long-lasting treatment for colorectal and breast cancer, is utilized in mono- and combination therapy for metastatic cancer (Johnston and Kaye, 2001). It is also used in preoperative therapy, neoadjuvant therapy and postoperative or adjuvant treatment with radiotherapy (Chan and Chee, 2019).

As capecitabine has become more widely used, severe adverse events have been reported, including gastrointestinal (GI) toxicities such as GI haemorrhage (Zou et al., 2021; Hagiwara et al., 2022), GI ulceration (Saeki et al., 2005), and GI obstruction (Pow-Anpongkul et al., 2019). Importantly, GI obstruction is not mentioned on the package insert. Therefore, the incidence of GI toxicity might be underestimated in clinical practice. Given the severity of this GI toxicity and the site of capecitabine absorption, physicians need to be aware of the profiles of drug toxicity, especially in populations at high risk.

Since 1969, the United States Food and Drug Administration Adverse Event Reporting System (FAERS) has been compiling data concerning adverse event (AE) reports to support the FDA’s safety surveillance of postmarketing drugs. Safety data on drug usage can be evaluated through data mining of FAERS (Revol et al., 2018). Data mining algorithms are broadly adopted to detect signals such as drug-associated AEs quantitatively. Pharmacovigilance analysis through FAERS is the most common way to identify rare signals and has potential value in novel signal detection (Meng et al., 2021).

## Aim

We aimed to determine the associations between capecitabine and GI ulceration, haemorrhage, and obstruction in patients with breast cancer by examining data from the FAERS.

## Materials and method

### Data acquisition and identification of AEs

This pharmacovigilance study was a retrospective disproportionality analysis of events in the FAERS database. We used OpenVigil to search for AE reports between 1 January 2004 and 31 December 2020. To avoid the impact of the primary disease on GI AEs, the AEs in this study were taken only from breast cancer patients. OpenVigil is a new publicly accessible pharmacovigilance tool for extracting, cleaning, mining, and dissecting data from FAERS using the interface of open-FDA (Bohm et al., 2012; Bohm et al., 2016).

All AE reports on FDA-approved capecitabine use that had the words “interacting,” “concomitant,” “primary suspect,” or “secondary suspect” were collected. All AEs in the FAERS are classified utilizing preferred terms (PTs) in the Medical Dictionary for Regulatory Activities (MedDRA, version 24.1). Based on our team’s previous experience with FAERS (Huang et al., 2022), we applied the definitions offered by MedDRA. The FAERS database collects information on AEs that are spontaneously reported by healthcare professionals, pharmaceutical manufacturers, patients, and others in different regions. Using MedDRA terms can overcome any interindividual inconsistencies in terminology by grouping like terms that are linked with an identical situation or AE of interest. Standardized MedDRA Queries (SMQs) are groupings of MedDRA terms broadly applied to FAERS research that can cover one defined medical situation or domain of interest (Meng et al., 2021). Disproportionality analysis was done to detect a relationship between exposure to capecitabine and the symptoms of GI ulceration, haemorrhage, or obstruction. The cases with any PT included in the broad scope of existing MedDRA SMQ terms (“Gastrointestinal ulceration,” “Gastrointestinal haemorrhage,” and “Gastrointestinal obstruction”) were chosen.

### Disproportionality analysis

Disproportionality analysis was employed to evaluate whether the reported number of a particular drug–AE combination exceeded the anticipated number. The “anticipated” number was the number of a given AE caused by other drugs in the database (Bate and Evans, 2010). Disproportionality analyses can be split into frequentist and Bayesian methods. We employed the information component (IC) and the reporting odds ratio (ROR) as the statistical gauges, based on a two-by-two contingency table, and the whole database was used as the comparison group. IC, a statistical gauge of the Bayesian method, is a tool for detecting signals that has the ability to avoid false positives, especially in a small number of cases (van Puijenbroek et al., 2002; Harpaz et al., 2013). ROR, a statistical gauge of the frequentist method, is the pharmacovigilance equivalent of the odds ratio and has been validated (Bate et al., 2014). A signal was present when the number of AEs was ≥3, the lower limit of the ninety-five percent confidence interval was >1 (van Puijenbroek et al., 2002), and the lower limit of the ninety-five percent confidence interval (IC025) of the IC value was >0 (Bate et al., 1998). When a positive signal was detected, we used sensitivity analysis to verify that the signal was stable. The sensitivity analytical conditions were age 65 years or older and a reporting time between 1 January 2011 and 31 December 2020.

### Statistical analysis

Microsoft Excel was used to perform descriptive analysis and assess the basic characteristics of the patients with capecitabine -associated GI ulceration, haemorrhage, and obstruction.

## Results

### AE reports and demographic characteristics

The search performed under the above conditions found 6,679 capecitabine -associated AEs in the FAERS (Table 1). Some 471 cases of GI ulceration, haemorrhage, and obstruction were detected, 94.90% of which occurred in female patients. The percentages of hospitalization, death, and life-threatening cases reached 52.23% (246/471), 25.05% (118/471), and 6.58% (31/471), respectively (Table 2).

**TABLE 1:** Features of capecitabine -associated AE reports submitted to the FAERS database.

	Capecitabine
Number of events	6,679
*Sex*	
Female	6,383 (96%)
Male	44 (1%)
Unknown	252 (4%)
*Age (years)*	
<18	1 (0%)
18–44	108 (2%)
45–64	2,723 (41%)
65–74	909 (14%)
≥75	414 (6%)
Unknown	2,524 (38%)
*Outcome of AE*	
Hospitalization (initial or prolonged)	1,956 (29%)
Disability	182 (3%)
Life-threatening	226 (3%)
Death	1,292 (19%)
Nonserious outcome^a^	3,554 (53%)

^a^ Nonserious outcomes refer to medical or health-related events that do not pose a significant risk to a patient’s life, health, or wellbeing. These outcomes are typically temporary, manageable, and do not require extensive medical intervention.

**TABLE 2:** Characteristics of gastrointestinal adverse events associated with capecitabine.

	Gastrointestinal ulceration	Gastrointestinal haemorrhage	Gastrointestinal obstruction
Number of events	290	126	55
*Sex*			
Female	276 (95%)	121 (96%)	50 (91%)
Male	0 (0%)	1 (1%)	0 (0%)
Unknown	14 (5%)	4 (3%)	5 (9%)
*Age (years)*			
<18	0 (0%)	0 (0%)	0 (0%)
18–44	8 (3%)	2 (2%)	0 (0%)
45–64	114 (39%)	59 (47%)	17 (31%)
65–74	58 (20%)	26 (21%)	15 (27%)
≥75	18 (6%)	10 (8%)	3 (5%)
Unknown	92 (32%)	29 (23%)	20 (36%)
*Outcome of AEs*			
Hospitalization (initial or prolonged)	119 (41%)	84 (67%)	43 (78%)
Disability	14 (5%)	3 (2%)	0 (0%)
Life-threatening	16 (6%)	9 (7%)	6 (11%)
Death	55 (19%)	45 (36%)	18 (33%)
Nonserious outcome^a^	144 (50%)	24 (19%)	11 (20%)

^a^ Nonserious outcome refer to medical or health-related events that do not pose a significant risk to a patient’s life, health, or wellbeing. These outcomes are typically temporary, manageable, and do not require extensive medical intervention.

### Disproportionality analyses

IC and ROR were calculated to find the signal values of GI ulceration, haemorrhage, and obstruction associated with capecitabine in the FAERS database. Compared to the overall AEs in the database, capecitabine -associated “gastrointestinal ulceration” [ROR 1.94 (1.71–2.21); IC 0.80 (0.60–0.99)], “gastrointestinal haemorrhage” [ROR 2.27 (1.86–2.76); IC 0.99 (0.69–1.28)], and “gastrointestinal obstruction” [ROR 2.19 (1.63–2.95); IC 0.96 (0.51–1.40)] had significantly higher reporting rates (Table 3). When the criteria for sensitivity analysis were “age 65 years or older” and “reporting time between 1 January 2011, and 31 December 2020,” the reporting rates of “gastrointestinal ulcers,” “gastrointestinal haemorrhage,” and “gastrointestinal obstruction” were significantly higher than those for all AEs (Table 3).

**TABLE 3:** Disproportionality analysis of capecitabine -associated GI ulceration, haemorrhage, and obstruction adverse events based on SMQ term.

	N	Label^a^	ROR	IC
Gastrointestinal ulceration	290	Y	1.94 (1.71–2.21)	0.80 (0.60–0.99)
Gastrointestinal haemorrhage	126	Y	2.27 (1.86–2.76)	0.99 (0.69–1.28)
Gastrointestinal obstruction	55	N	2.19 (1.63–2.95)	0.96 (0.51–1.40)
Age 65 years or older				
Gastrointestinal ulceration	77	Y	2.53 (1.96–3.27)	1.11 (0.74–1.49)
Gastrointestinal haemorrhage	36	Y	2.79 (1.92–4.06)	1.25 (0.69–1.80)
Gastrointestinal obstruction	18	N	3.27 (1.91–5.57)	1.43 (0.64–2.22)
Report between 1 January 2011 and 31 December 2020				
Gastrointestinal ulceration	155	Y	1.46 (1.23–1.74)	0.47 (0.22–0.73)
Gastrointestinal haemorrhage	50	Y	1.47 (1.09–1.98)	0.49 (0.05–0.93)
Gastrointestinal obstruction	26	N	1.72 (1.13–2.61)	0.69 (0.07–1.31)

^a^ Whether the AEs, are listed on drug labels or not.

## Discussion

This study found that capecitabine may cause GI ulceration, haemorrhage, and obstruction. Gastrointestinal obstruction was not mentioned in the package insert and can be considered a new potential adverse effect with value in guiding clinical care.

Early studies revealed that capecitabine causes mitotic arrest of crypt cells in the G2 phase, impairing their migration and differentiation into mature enterocytes (Ikuno et al., 1995). In addition, fluoropyrimidines may increase the gene expression of inflammatory cytokines and reduce the expression of colonic aquaporin channels through neutrophilic inflammation (Sakai et al., 2013). Both pathways induce intestinal inflammation by inducing epithelial cell loss secondary to acute mucosal injury. Pete Pow-Anpongkul reported (Pow-Anpongkul et al., 2019) on a 60-year-old man with colorectal cancer who presented with abdominal pain, bloating, and vomiting after two cycles of capecitabine. Abdominal CT showed thickening of the small bowel wall involving the distal duodenum and jejunum with dilated gastric and proximal small bowel loops, consistent with the diagnosis of ileus. However, endoscopy revealed severe inflammation from the distal duodenum to the proximal jejunum. Mild enteritis may be characterized by abdominal pain and diarrhoea (Gómez-Escudero and Remes-Troche, 2021; Trontzas et al., 2023), while severe enteritis may lead to intestinal ulceration and obstruction (Ali and Robles, 2021; Hamdeh et al., 2021; Sasaki et al., 2022).

Fluoropyrimidines are directly toxic to the endothelium through the increased generation of reactive oxygen species. This toxicity can cause vasospasm or thrombosis through the release of sequestered vasoactive substances (Focaccetti et al., 2015). If mesenteric blood vessels are embolized, the intestinal blood circulation will be impaired, and the intestinal tube will lose its peristaltic ability, thereby leading to intestinal vascular obstruction; this phenomenon explains the intestinal obstruction caused by capecitabine (Stancu et al., 2024). Obstruction might also be due to patient factors. Capecitabine is converted to 5-FU in the body, and most 5-FU is catabolized by dihydropyrimidine dehydrogenase (DPD) (Matsusaka and Lenz, 2015). DPD deficiency is reported in roughly 5%–9% of patients, so administering fluoropyrimidines in the context of depressed enzyme activity can be fatal (Saif and Diasio, 2006; Wormann et al., 2020). We speculate that DPD deficiency aggravates the gastrointestinal toxicity caused by capecitabine and increases the likelihood of gastrointestinal haemorrhage, ulceration, and obstruction. Of course, a combination of factors could lead to GI obstruction in patients taking capecitabine.

Recommendations are in place for routine screening for the four most common DPD variants before initiating treatment with capecitabine (Iacovelli et al., 2014). For patients with DPD deficiency, a Japanese study showed that the initial administration of a small amount of capecitabine followed by a gradual dose increase could eventually increase the DPD protein level up to 12-fold before chemotherapy. This safe administration method for patients with DPD deficiency is worth exploring in clinical practice (Yoshida et al., 2015).

Another approach that deserves attention is probiotics. Probiotics may have antitumour effects, particularly against colorectal and breast cancer (de Moreno de LeBlanc et al., 2007; Osterlund et al., 2007), and are more suitable for patients with breast or colorectal cancer who are treated with capecitabine. Recent studies on inflammatory bowel disease have revealed a pivotal therapeutic and anti-inflammatory role for probiotics (Sartor and Muehlbauer, 2007). Potential mechanisms of probiotic action include competitive inhibition of pathogen binding to intestinal epithelial cells, inhibition of pro-inflammatory cytokines, induction of anti-inflammatory cytokines, production of antimicrobial metabolites, dialogue with the epithelium, and immune modulation (de Moreno de LeBlanc et al., 2007; Osterlund et al., 2007). Probiotics may play a preventive role in ileitis-induced obstruction.

Patients should be adequately educated on medication safety. Intestinal obstruction is a surgical emergency in the abdomen. If early detection and timely treatment are performed, satisfactory clinical results can be achieved. Otherwise, the treatment will possibly cause delay, and in addition, complications can occur, such as abdominal infection and septic shock which can be fatal. Therefore, medical staff should fully inform patients who take capecitabine about the health issues reported for this medication.

This research has some limitations 1) Overreporting, missing figures, and underreporting were unavoidable owing to the voluntary and retrospective nature of FAERS reporting (Michel et al., 2017). 2) Considering the possibility of confounding elements, including concomitant drugs or potential illnesses, causal correlations cannot be definitively inferred from our results. 3) The IC or ROR does not indicate an increased risk of AE occurrence but rather an increased risk of AE reports (Raschi et al., 2020). 4) Due to database limitations, we do not have a clear picture of whether patients were taking capecitabine in combination with radiotherapy and whether there were gene variants in the DPYD gene. It is not possible to determine whether these factors are associated with severe gastrointestinal toxicity. 5) The only disease examined in this study was breast cancer, and although the positive signal was obtained, it does not indicate that the relevant results may occur in other diseases. The above limitations also lead to the difference between the incidence of the data collected in this study and the drug label. The nature of FAERS weakens the conclusions that can be drawn. Nevertheless, this research offers a pivotal foundation for hypothesis generation (but not testing) and emphasizes possible patient health problems worthy of thorough exploration.

Despite these limitations, our research is the first to recognize the link between capecitabine and GI ulceration, haemorrhage, and obstruction by analysing the world’s most extensive database of postmarketing drugs. Our findings will contribute to the safe utilization of capecitabine. To further study whether capecitabine causes gastrointestinal toxicity in patients with colorectal cancer, we retrospectively analysed data from three medical institutions that have given patients capecitabine monotherapy after colorectal cancer surgery over the past 10 years. The investigation is ongoing, and we will publish our findings in due course. In addition, more prospective and preclinical epidemiological research is needed to verify our findings and conclusions.

## Conclusion

By searching the FAERS database, we found an adverse reaction, GI obstruction, that was not listed on the capecitabine drug label. The present pharmacovigilance research revealed a mild increase in reports of GI ulceration, haemorrhage, and obstruction, which might trigger severe and even fatal outcomes, among capecitabine users. Further in-depth epidemiological research is needed to validate any causal relationships.

## Impact statements

Significant increases were observed in the reported rates of “gastrointestinal ulceration,” “gastrointestinal haemorrhage,” and “gastrointestinal obstruction” associated with capecitabine. GI obstruction was found to be a positive adverse reaction signal not recorded in the package insert.

Although no clinical trials or observational studies have confirmed these signals, capecitabine’s medication guidance should be reinforced to warn of GI toxicity, including GI ulceration, haemorrhage, and obstruction.

More studies on capecitabine are needed to characterize its GI ulceration, haemorrhage, and obstruction risk factors and outcomes.

## Data Availability

The raw data supporting the conclusion of this article will be made available by the authors, without undue reservation.
